# Weighted Gene Co-expression Network Analysis of Key Biomarkers Associated With Bronchopulmonary Dysplasia

**DOI:** 10.3389/fgene.2020.539292

**Published:** 2020-09-09

**Authors:** Yao Cai, Fei Ma, LiuHong Qu, Binqing Liu, Hui Xiong, Yanmei Ma, Sitao Li, Hu Hao

**Affiliations:** ^1^Department of Neonatology, The Sixth Affiliated Hospital of Sun Yat-sen University, Guangzhou, China; ^2^Department of Neonatology, The Maternal and Child Health Care Hospital of Huadu, Guangzhou, China; ^3^Huadu Affiliated Hospital of Guangdong Medical University, Guangzhou, China; ^4^Laboratory of Inborn Metabolism Errors, The Sixth Affiliated Hospital of Sun Yat-sen University, Guangzhou, China

**Keywords:** bronchopulmonary dysplasia, weighted gene co-expression network analysis, hub gene, biological process, biomarkers

## Abstract

Bronchopulmonary dysplasia (BPD) is a complex disorder resulting from interactions between genes and the environment. The accurate molecular etiology of BPD remains largely unclear. This study aimed to identify key BPD-associated genes and pathways functionally enriched using weighted gene co-expression network analysis (WGCNA). We analyzed microarray data of 62 pre-term patients with BPD and 38 pre-term patients without BPD from Gene Expression Omnibus (GEO). WGCNA was used to construct a gene expression network, and genes were classified into definite modules. In addition, the Gene Ontology (GO) and Kyoto Encyclopedia of Genes and Genomes (KEGG) analyses of BPD-related hub genes were performed. Firstly, we constructed a weighted gene co-expression network, and genes were divided into 10 modules. Among the modules, the yellow module was related to BPD progression and severity and included the following hub genes: *MMP25*, *MMP9*, *SIRPA*, *CKAP4*, *SLCO4C1*, and *SLC2A3*; and the red module included some co-expression molecules that displayed a continuous decline in expression with BPD progression and included the following hub genes: *LEF1*, *ITK*, *CD6*, *RASGRP1*, *IL7R*, *SKAP1*, *CD3E*, and *ICOS*. GO and KEGG analyses showed that high expression of inflammatory response-related genes and low expression of T cell receptor activation-related genes are significantly correlated with BPD progression. The present WGCNA-based study thus provides an overall perspective of BPD and lays the foundation for identifying potential pathways and hub genes that contribute to the development of BPD.

## Introduction

Bronchopulmonary dysplasia (BPD) is a chronic lung disease in pre-term infants that is characterized by arrested lung development due to early lung injury (Jobe, 1999; Speer, 2006a). Since its first description in Northway et al. (1967), with the survival of increasing number of premature babies having very low birth weights, the incidence of BPD has remained high. However, few specific treatments are available for reducing the burden of the disease (Lemons et al., 2001; Farstad et al., 2011). Survivors of BPD have an increased risk of pulmonary hypertension, growth retardation, neurodevelopmental delay, and other long-term sequelae that have a major impact on families and health care system (Bhandari and McGrath, 2013). However, the mechanism of BPD formation is complex and includes many processes, such as inflammation, oxidative stress, abnormal angiogenesis, and damaged lung repair. Some of these processes remain to be elucidated (Coalson, 2003; Chess et al., 2006; Collins et al., 2017; Yang et al., 2017).

Weighted gene co-expression network analysis (WGCNA) is an algorithm that defines modules of genes with similar expression patterns in complex diseases (Langfelder and Horvath, 2008). WGCNA can effectively integrate gene expression and clinical trait data to appraise functional pathways and candidate molecular biomarkers (Presson et al., 2008). WGCNA facilitates a global interpretation of gene expression data through the construction of gene networks based on the similarity of expression profiles among samples (Oldham et al., 2008). WGCNA has been used for the study of gene-network signatures, co-expression modules, and hub genes involved in human respiratory syncytial virus infection (Vieira et al., 2019), autoimmune diseases (Medina and Lubovac-Pilav, 2016; Ma et al., 2017), and various cancers (Giulietti et al., 2016; Tian et al., 2019). A hub gene is a key gene that plays a vital role in regulatory pathways; the regulation of other genes is often affected by this gene (Luscombe et al., 2004). Thus, WGCNA can be used to elucidate gene-network signatures and hub genes associated with BPD to better understand the pathogenesis of this disease. To our knowledge, however, WGCNA, as a system biology approach, has not been applied to the analysis of BPD-derived data thus far.

In the present study, we used WGCNA to explore the gene-network signatures of peripheral blood from pre-term infants with and without BPD. The pathogenesis of BPD was explored using pathway enrichment analysis to investigate the biological pathways and key hub genes that were associated with BPD. Finally, enrichment analysis was used to determine the potential functions of these hub genes and to identify key genes potentially involved in the pathogenesis of BPD.

## Materials and Methods

### Data Collection and Preprocessing

**FIGURE 1:**
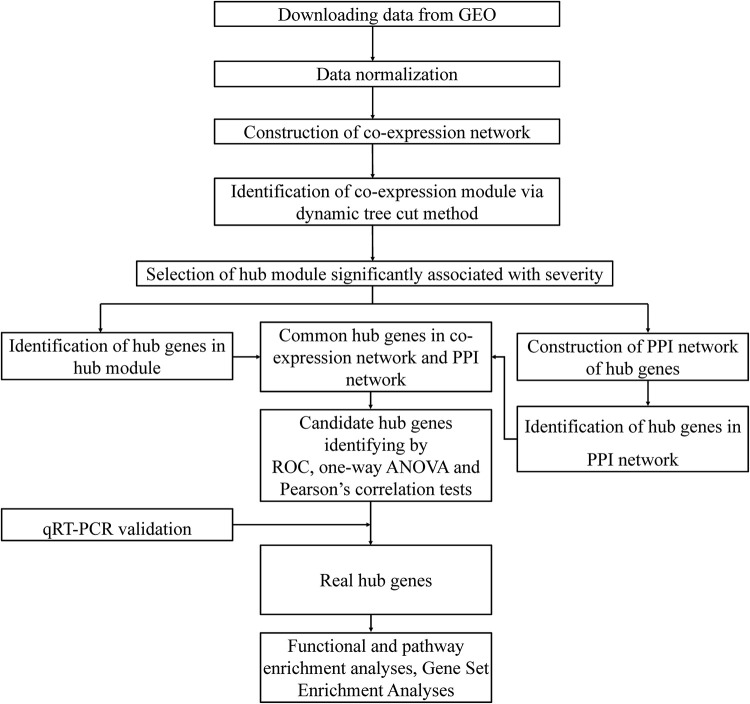
Outline of the study design.

A flow chart illustrating the data preparation, processing, and analysis is displayed in Figure 1. We used ‘bronchopulmonary dysplasia’ as the key word to search the Gene Expression Omnibus (GEO) database and to select datasets containing samples from different pathological stages and normal controls. Finally, the dataset GSE32472^1^, was found to meet our requirements and was therefore downloaded. To identify the molecular networks and hub genes related to the pathological progress of BPD, WGCNA was conducted. GSE32472 provided microarray profiles of blood samples of newborns with BPD, including microarray assessment of gene expression at approximately the 5th, 14th, and 28th days of life. To ensure the stability of the selection, 100 blood samples at about the 28th day were selected, when a more definite diagnosis of BPD had been made. These samples were obtained from 38 controls, and 38 mild, 10 moderate, and 14 severe BPD cases. The expression data were normalized using quantile normalization function in limma package of R software (Ritchie et al., 2015). The genes with the highest variance in expression values (top 25%) were selected for co-expression network construction. Cluster analysis using the Pearson’s correlation matrices and the average linkage method were conducted to detect whether outlier samples existed for the purpose of ensuring the reliability of the network construction. A brief design of the study is shown in Figure 1A.

### Construction of a Co-expression Network

A co-expression network was constructed using the WGCNA algorithm package in R (Presson et al., 2008). First, the Pearson’s correlation matrices were constructed for all pair-wise genes. Next, a weighted adjacency matrix was constructed by using the power function amn = | Cmn| ^β^ (Cmn = Pearson correlation between gene m and gene n; a_mn_ = adjacency between gene m and gene n) (Zhang and Horvath, 2005). The parameter β served as a soft threshold parameter to expand strong correlations and penalize weak correlations between genes. To ensure a scale-free topology of the network, β was selected when the scale independence value was equal to 0.9. The adjacency was transformed into a topological overlap matrix (TOM) to measure the network connectivity of a gene, which is defined as the sum of its adjacency with all other genes. Hierarchical clustering was performed according to TOM-based dissimilarity to distribute genes with similar expression patterns into modules with a minimum cluster size of 50 (Ravasz et al., 2002). Highly similar modules were merged with a cut-off of 0.25.

### Identification of Modules Significantly Associated With BPD Severity

Module eigengenes (MEs) were considered the major component in the principal component analysis for each gene module, and the expression patterns of all genes could be summarized into a single characteristic expression profile within a given module. To identify modules significantly associated with BPD severity, the correlation between MEs and BPD stage was evaluated by the Pearson’s correlation test with *p* < 0.05 as the cut-off. The modules most significantly related to BPD severity were considered as key modules and subjected to further analysis.

### Identification of Candidate Hub Genes

A module hub gene is a highly connected in-module gene that has the highest module member (MM) score of its corresponding module (Horvath and Dong, 2008). The MM score for every gene was calculated by the WGCNA function KME, which correlates the expression profile of a gene with the ME of a module to quantify the relationship between a gene and a given module. The absolute value of gene significance (GS) represents the Pearson’s correlation between a given gene and clinical features. We removed hub genes based on the cut-off criteria (| MM| ≥ 0.85, | GS| ≥ 0.45). Further, all genes in key modules were uploaded to STRING^2^ to acquire information about the interaction between genes. Protein–protein interaction (PPI) networks were constructed with the species limited to ‘*Homo sapiens*’ and a confidence > 0.9. In the PPI network, genes with a degree ≥ 10 were defined as hub nodes. Hub genes common in both co-expression network and PPI network were selected for candidate hub genes identification.

Hub genes common in both co-expression network and PPI network were analyzed by ROC curve, and area under the curve (AUC) was calculated to distinguish the control group from the BPD group. In addition, one-way ANOVA and Pearson’s correlation tests were conducted to explore the relevance of the hub genes common in both co-expression network and PPI network in terms of disease severity. Candidate hub genes were identified using the following criteria: (1) a significant *P* value in the one-way ANOVA test and the Pearson’s correlation and (2) an AUC > 0.8.

### qRT-PCR Validation and Real Hub Genes Identification

To validate the candidate hub genes obtained by WGCNA, pre-term infants with or without BPD blood samples were collected from the Department of Neonatology of the Sixth Affiliated Hospital of Sun Yat-sen University. This research was approved by the ethics review board of Sixth Affiliated Hospital of Sun Yat-sen University (2019ZSLYEC-80), and written informed consent was provided by the participants’ legal guardians. From each sample, 100 ng of cDNA was obtained for RT-PCR amplification reaction, and the expression of an endogenous control (housekeeping gene: GAPDH) was used for the determination of the relative expression levels of the hub genes. Primer sequences for related hub genes are listed in Supplementary Table S1. Real hub genes were identified if the results of RT-PCR have significant difference.

### Functional and Pathway Enrichment Analyses

To gain further insights into the functions of hub genes in the module most related to BPD, we performed biological process analysis and KEGG pathway enrichment analysis with ‘c2.cp.kegg.v7.1.symbols’ as background^3^.

### Gene Set Enrichment Analysis (GSEA) for Hub Biological Pathways Confirmation

Mapping to KEGG (Kyoto Encyclopedia of Genes and Genomes) database^4^, GSEA^5^ (Subramanian et al., 2007) was performed between control and BPD groups to confirm the expression pattern of hub biological pathways.

### Statistical Analysis

Non-parametric tests or *t*-tests based on data distribution characteristics were used to analyze the statistical significance of the difference in hub gene expression levels between the two groups. Analyses were conducted in GraphPad Prism 8.0.2. *P* < 0.05 was considered statistically significant.

## Results

### Weighted Co-expression Network Construction

**FIGURE 2:**
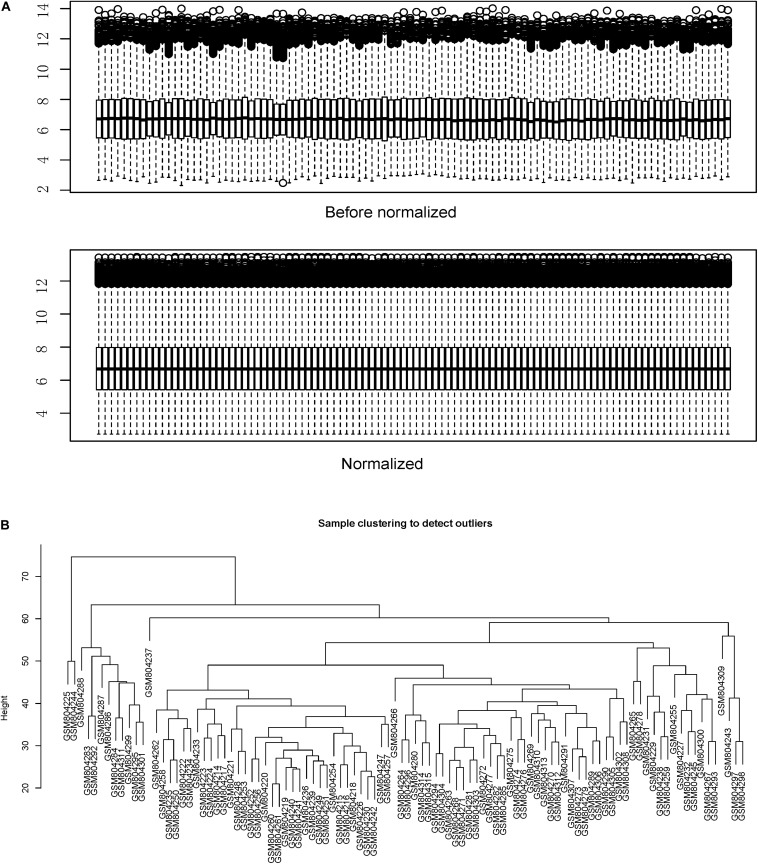
Data normalization and sample clustering dendrogram. **(A)** Data were normalized using the limma package of the R software. **(B)** Sample clustering was performed using the Pearson’s correlation matrices and the average linkage method.

**FIGURE 3:**
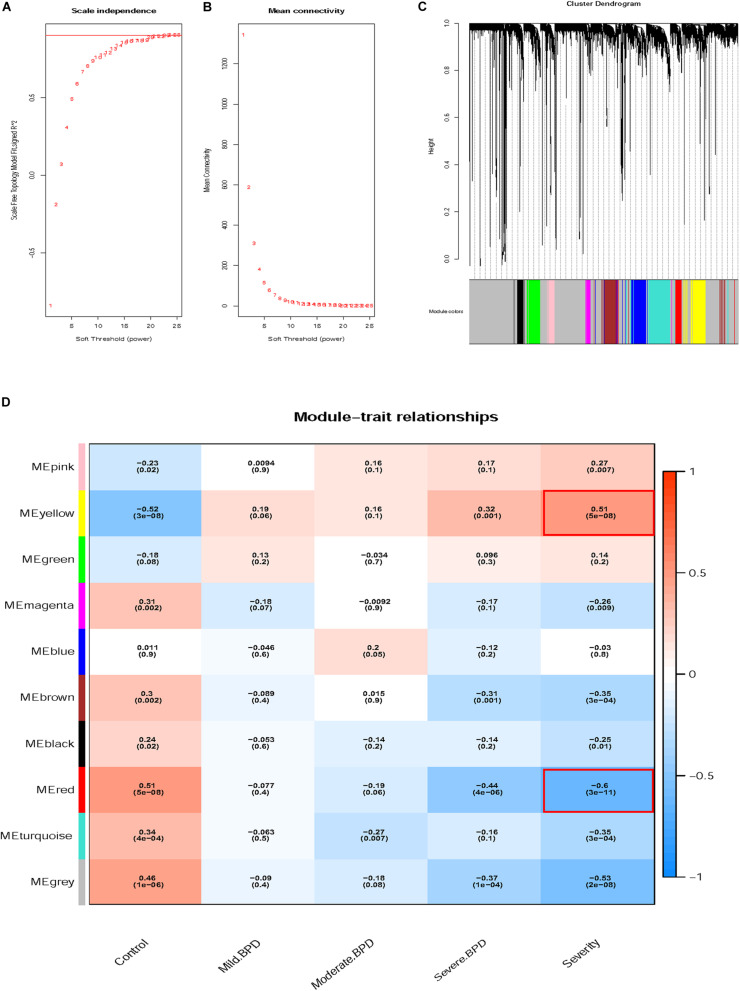
Determination of soft-thresholding power and grouping of genes with similar expression into modules using weighted gene co-expression network analysis (WGCNA). **(A)** Analysis of the scale-free fit index for soft-thresholding powers (β). **(B)** Analysis of the mean connectivity for soft-thresholding powers. **(C)** Dendrogram of clustered genes. **(D)** Identification of modules associated with clinical information.

Figure 1 shows the flow chart of data preparation, processing, analysis, and validation for this study. The data were normalized using the limma package of R software (Figure 2A and R code in Supplementary Table S2). The co-expression analysis included 100 samples with clinical information, sample information, and expression matrix. Input files are provided in Supplementary Tables S3, S4. Sample clustering was performed based on Pearson’s correlation matrices and the average linkage method. No outliers were detected (Figure 2B). The genes showing the highest expression variance (top 25%) were selected for subsequent WGCNA using the WGCNA package in R software. Genes with similar expression patterns were then grouped by average linkage hierarchical clustering. In our study, β = 23 (scale-free *R*^2^ > 0.901) was selected as the soft threshold to ensure a scale-free network (Figures 3A,B). Next, we constructed a systematic clustering tree using the WGCNA package. In Figure 3C, each short vertical line represents a gene, and each color represents one module composed of genes with similar expression patterns. The genes shown in gray were the genes that could not be merged. A total of 10 modules were identified (Figure 3C).

### Identification of Key Modules Associated With BPD Severity

We tested the relevance of each module for BPD clinical information, focusing on different BPD stages. As displayed in Figure 3D, the yellow module (*P* = 5e-08, *R*^2^ = 0.51) was most significantly and positively correlated with BPD severity, whereas the red module (*P* = 3e-11, *R*^2^ = −0.60) showed the opposite result. The correlation between the yellow module and BPD severity gradually increased and finally became positive. The red module showed the opposite pattern. Based on the above findings, the red and yellow modules were identified as key modules correlated with BPD severity and were thus, further analyzed.

### PPI Network Construction With Corresponding Module Genes

**FIGURE 4:**
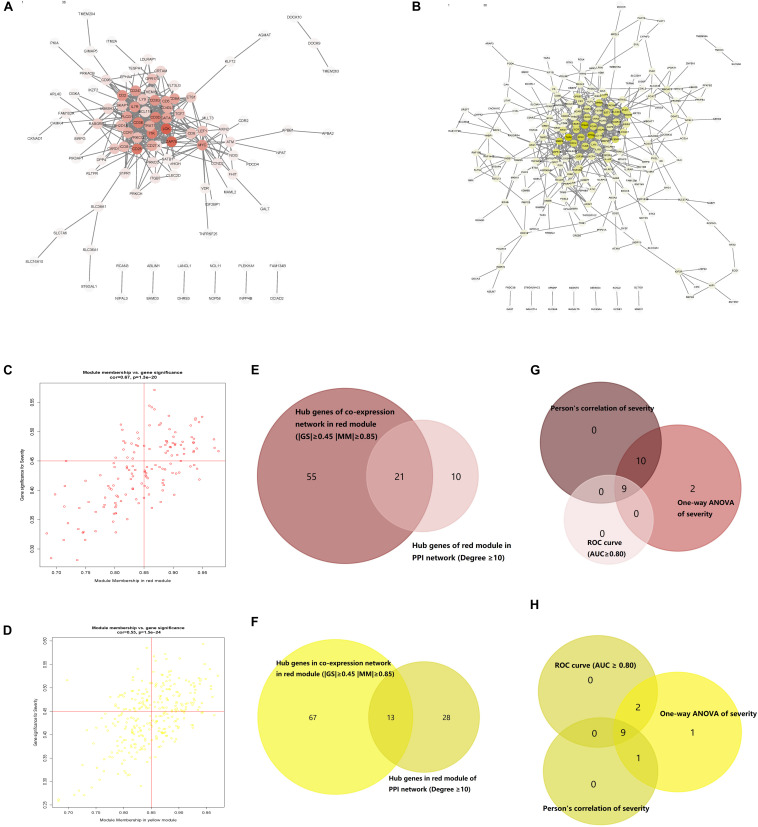
Protein–protein interaction (PPI) networks of genes corresponding to the two key modules. **(A)** PPI network of the nodes in the red module. **(B)** PPI network of the nodes in the yellow module. **(C)** Scatter plot of module eigengenes (MEs) in the red module. **(D)** Scatter plot of MEs in the yellow module. **(E)** Common red hub genes in the co-expression and PPI networks. **(F)** Common yellow hub genes in the co-expression and PPI networks. **(G)** Common genes in the red module shared characteristics with an area under the curve (AUC) ≥ 0.80 and had significant *P* values in the Pearson’s correlation and one-way ANOVA tests. **(H)** Common genes in the yellow module shared characteristics with an AUC ≥ 0.80 and had significant *P* values in the Pearson’s correlation and one-way ANOVA tests.

PPI networks of the red and yellow modules were constructed with a cutoff confidence > 0.9 (Figures 4A,B). A total of 31 genes in the red module and 41 genes in the yellow module were identified with a degree ≥ 10 as hub genes in each PPI network. Based on | MM| ≥ 0.85 and | GS| ≥ 0.45, a total of 76 genes in the red module and 77 genes in the yellow module were selected as hub genes in each co-expression network (Figures 4C,D). A total of 21 genes in the red module and 13 genes in the yellow module were identified in both the PPI and co-expression networks (Figures 4E,F). All GS, MM, and intramodule connectivity values of each identified module are listed in Supplementary Tables S5–S7.

### Identification of Real Hub Genes

**FIGURE 5:**
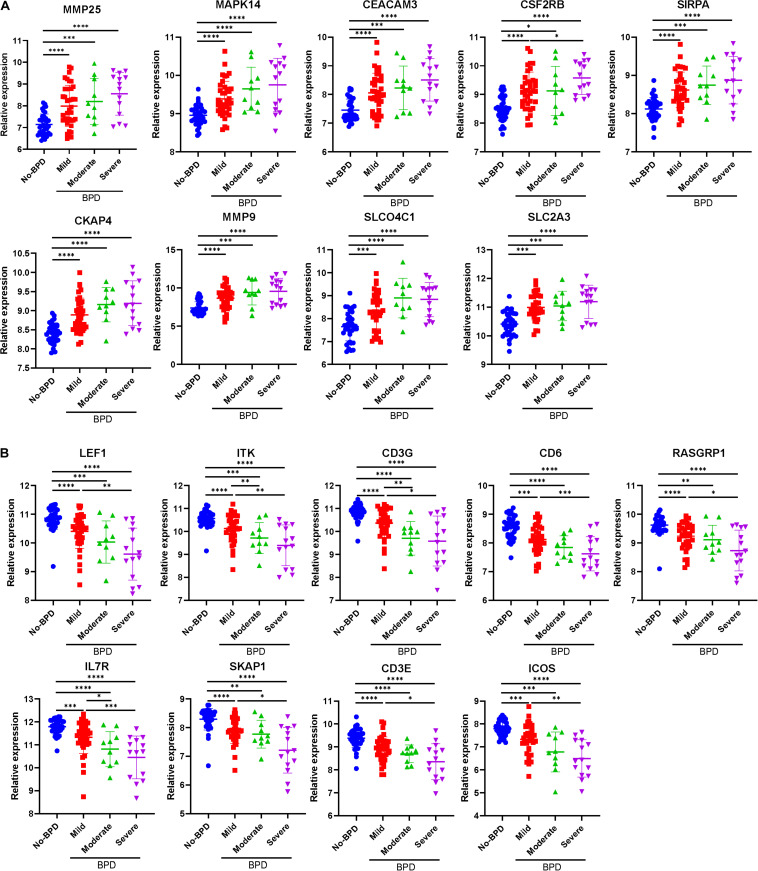
Severity plot of the real hub genes. **(A)** Severity plot of the identified hub genes in the yellow module. **(B)** Severity plot of the identified hub genes in the red module. *P < 0.05, **P < 0.01, ***P < 0.001, and ****P < 0.0001.

**FIGURE 6:**
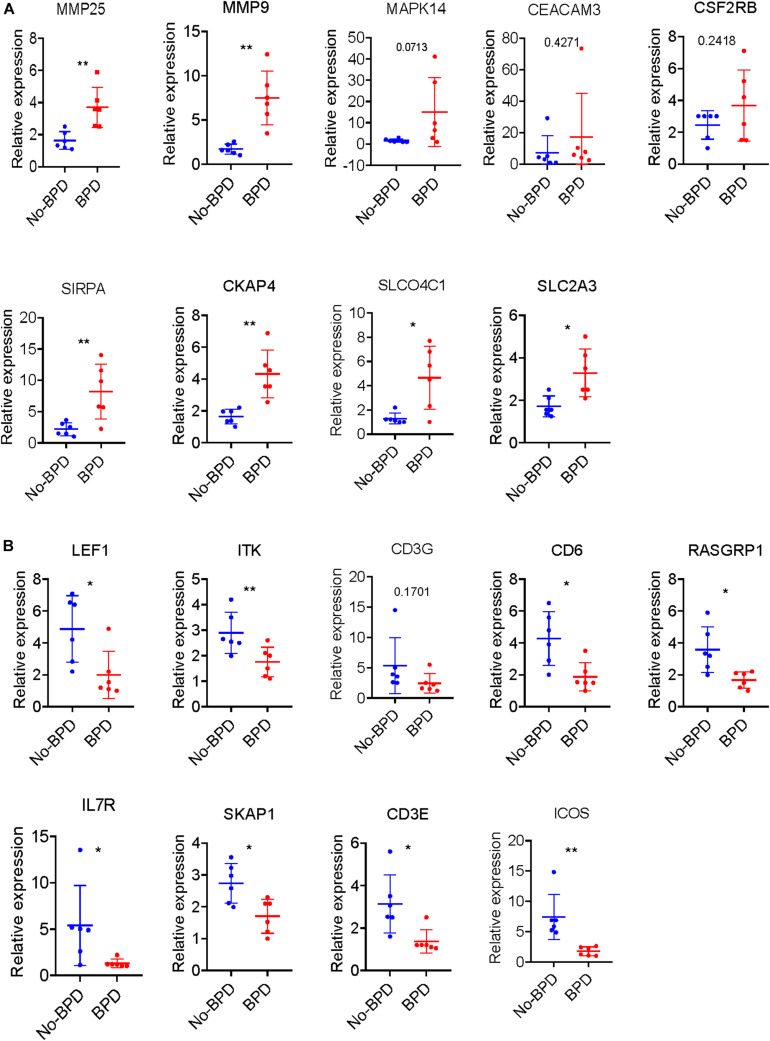
Validation of hub genes by qRT-PCR. **(A)** Severity plot of the identified hub genes in the yellow module analyses by qRT-PCR. **(B)** Severity plot of the identified hub genes in the red module analyses by qRT-PCR. *P < 0.05 and **P < 0.01.

All the 21 candidate hub genes in the red module showed significance in the one-way ANOVA. A total of nine genes had an AUC ≥ 0.80, and 19 showed a significant correlation with disease severity in the Pearson’s correlation analysis. Eventually, nine genes in the red module with an AUC ≥ 0.80 and significant *P* values in the Pearson’s correlation as well as one-way ANOVA were regarded as candidate hub genes (Figure 4G). Similarly, of the 13 candidate hub genes in the yellow module, 12 showed significance in the one-way ANOVA, 11 genes had an AUC ≥ 0.80, and 10 showed a significant correlation with disease severity in the Pearson’s correlation analysis. Nine genes in the yellow module had an AUC ≥ 0.80 and significant *P* values in the Pearson’s correlation as well as in the one-way ANOVA, and were thus, selected as candidate hub genes (Figure 4H). Detailed information about the red and yellow modules in relation to the Pearson’s correlation, ROC, and one-way ANOVA has been provided in Supplementary Tables S8–S11. The severity plot for the candidate hub genes is shown in Figure 5A. The expression levels of candidate hub genes in the yellow module increased with disease severity, and the expression levels of candidate hub genes in this module were significantly increased in different BPD severity conditions compared with those of normal controls. In contrast, candidate hub genes in the red module showed decreasing expression levels with greater disease severity, and markedly decreased expression levels in different BPD severity conditions (Figure 5B). To further clarify the clinical significance and identify real hub genes, we collected the BPD patient’s blood for qRT-PCR validation *in vitro*. The results showed that most of these genes had statistically significant differences and were considered as real hub genes, except for MAPK14, CEACAM3, CSF2RB, and CD3G (Figure 6).

### Functional and Pathway Enrichment Analysis

**FIGURE 7:**
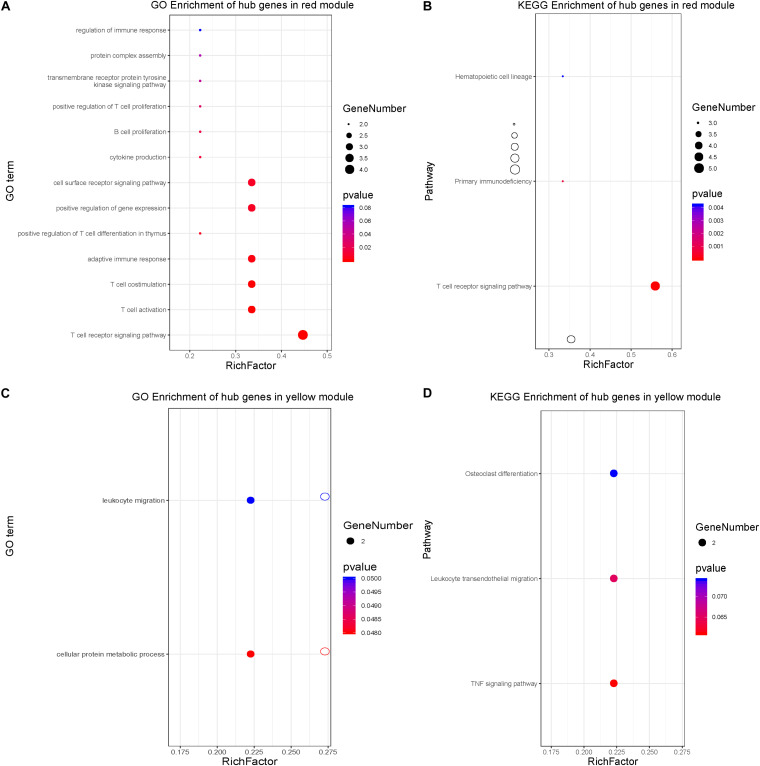
Biological process and KEGG pathway enrichment analyses of hub genes. **(A)** Biological process analysis for the hub genes in the red module. **(B)** KEGG pathway enrichment for the hub genes in the red module. **(C)** Biological process enrichment for the hub genes in the yellow module. **(D)** KEGG pathway enrichment for the hub genes in the yellow module.

To learn more about the function of the identified hub genes, they were subjected to perform the biological process and KEGG pathway enrichment analyses. Real hub genes in the red module, which exhibited a negative correlation with disease severity, were significantly enriched in 20 BPs: T cell activation, positive regulation of leukocyte cell-cell adhesion, positive regulation of cell-cell adhesion, regulation of leukocyte cell-cell adhesion, leukocyte cell-cell adhesion, positive regulation of cell adhesion, regulation of cell-cell adhesion, positive regulation of T cell activation, regulation of cell-cell adhesion, positive regulation of T cell activation, positive regulation of lymphocyte activation, positive regulation of leukocyte activation, regulation of T cell activation, positive regulation of cell activation, regulation of lymphocyte activation, regulation of leukocyte activation, T cell differentiation, lymphocyte differentiation, interleukin-4 production, T cell differentiation in thymus, positive regulation of T cell differentiation in thymus, and regulation of cell-cell adhesion mediated by integrin (Figure 7A). The real hub genes were also enriched in three KEGG pathways: T cell receptor signaling pathway, primary immunodeficiency, and hematopoietic cell lineage (Figure 7B). The real hub genes in the yellow module, which showed a positive correlation with disease severity, were enriched in 4 BPs, neutrophil degranulation, neutrophil activation involved in immune response, neutrophil mediated immunity, and neutrophil activation (Figure 7C). KEGG pathway enrichment analysis showed that real hub genes in the yellow module were enriched in the bladder cancer pathway, and leukocyte transendothelial migration (Figure 7D).

### Gene Set Enrichment Analysis for Hub Biological Pathways Confirmation

**FIGURE 8:**
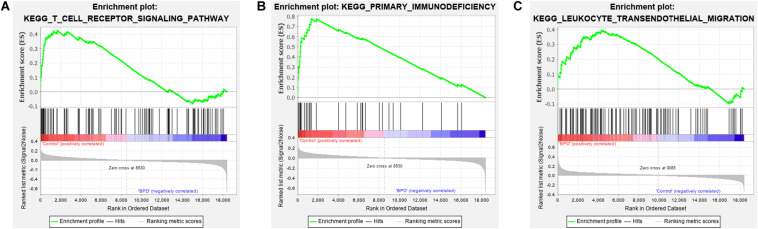
Gene set enrichment analysis of real hub genes. **(A)** Enrichment result of ‘T cell receptor signaling pathway’ between the control group and the bronchopulmonary dysplasia (BPD) group. **(B)** Enrichment result of the term ‘primary immunodeficiency’ between the control group and the BPD group. **(C)** Enrichment result of ‘leukocyte transendothelial migration’ pathway between the control group and the BPD group.

As bladder cancer pathway is not related to BPD, it was not considered in GSEA confirmation. According to the results of the GSEA, the terms ‘T cell receptor signaling pathway’ and ‘primary immunodeficiency’ were significantly enriched in the control group while the term ‘hematopoietic cell lineag’ was not (Figures 8A,B and Supplementary Table S12). On the contrary, the term ‘leukocyte transendothelial migration’ was significantly enriched in the BPD group (Figure 8C and Supplementary Table S13). These results successfully confirmed the expression pattern of hub biological pathways.

## Discussion

To our knowledge, our study reports the first application of WGCNA to construct a BPD-related gene-network. We found two key gene modules and several hub genes that were associated with BPD progression. This research provides new insights into the molecular etiology of BPD, as well as potential therapeutic targets for this disease. Ten co-expression modules were obtained through WGCNA. The yellow module was associated with progression and severity of BPD and the red module included co-expressed genes that displayed a continuous decline in expression with BPD progression.

Among the 10 modules, the yellow module was especially involved in BPD pathogenesis. Some genes showed greater positive association with the progression of BPD including *MMP25*, *MMP9*, *SIRPA*, *CKAP4*, *SLCO4C1*, and *SLC2A3*. The red module contained genes showing greater negative association with the progression of BPD including *LEF1*, *ITK*, *CD6*, *RASGRP1*, *IL7R*, *SKAP1*, *CD3E*, and *ICOS*. These genes can be considered as hub genes and also play important roles in other co-expression modules.

Functional enrichment analysis is widely used to classify biological entities into functionally related groups (Rue-Albrecht et al., 2016). In the present study, we used the GO and KEGG analyses to elucidate the biological functions of hub genes in the yellow module, that were significantly up-regulated with the increase of BPD severity. The genes in the yellow module were mainly enriched in the response to cellular protein metabolic processes, leukocyte migration, and TNF signaling pathway. The inflammatory response plays critical roles in the development of BPD (Shahzad et al., 2016). Consistent with previous reports (Ma et al., 2017), we found a significant increase in levels of *MMP9* and *MMP25* in infants with BPD compared with those in infants without BPD. This consistency not only further demonstrates the reliability of our results, but also provides additional confirmation of the pivotal role of MMP proteins in BPD progression. Disease-related gene expression analysis revealed signaling pathways involved in BPD progression, including protein kinase A, MAPK, and neuromodulin/epidermal growth factor receptor signal. In a newborn Sprague-Dawley rat BPD model, activation of the MAPK and PI3K/AKT signaling pathways in lung tissues was monitored during prolonged exposure of newborn rats to hyperoxia (Liu et al., 2018). This previous study suggested that MAPK14 could be used as a biological marker to monitor disease progression.

The most notable down-regulated pathway in BPD progression is the T cell receptor signaling pathway. Our data showed that the expression of T cell receptor molecules, including *CD3E*, *CD6*, and *ICOS*, decreased significantly during BPD progression. These molecules had not been confirmed in previous studies. T cell response depends on the type of ligand that binds to the receptor, the duration of cooperation, and the presence of co-receptors or co-inhibitors (Cheng et al., 2011; James et al., 2011). In our study, transcription factors and related pathways, such as *CD3E*, *CD6*, and *ICOS*, were under-expressed in children with BPD, suggesting that reduced T receptor expression may lead to decreased receptor density at the cell surface, which in turn may be a risk factor for bacterial translocation and further infection. These results are consistent with the fact that pulmonary infection is a risk factor for BPD (Speer, 2006b).

Enrichment analysis revealed the signaling pathways that may be related to the pathogenesis of the disease. The results can be considered in two ways. One is by placing our findings in the context of the existing knowledge, and the other is by studying genes known to be potentially involved in the pathogenic mechanism of BPD. The overexpression of pathways involved in inflammatory cytokine production and leukocyte migration in the present study confirms the generally accepted contribution of inflammatory responses to the etiology of BPD. By contrast, we found a low expression of genes related to other immune response pathways, including the T cell receptor pathway. Pietrzyk et al. (2013) reported that overexpression of pathways involving cytokines and their receptors confirms the widely accepted role of inflammatory responses in the etiology of BPD, and that T cell response pathways are closely related to infant maturity (Pietrzyk et al., 2013). Therefore, based on the above-mentioned research studies, our research has revealed more specific regulatory molecules to provide new targets for the prediction of BPD and for targeted interventions.

In summary, this study applied WGCNA to a large dataset to explore BPD-related co-expression gene networks. Our results revealed the roles of key co-expression module genes, hub genes, and functional biological pathways were associated with the down-regulation of the T cell receptor signaling pathway, the enrichment of the TNF signaling pathway and leukocyte migration in BPD pathogenesis, thus providing new insights into the development of BPD. However, the exact molecular mechanisms connecting hub genes and functional pathways of BPD need further exploration.

## Data Availability Statement

All datasets generated for this study are included in the article/Supplementary Material.

## Ethics Statement

The studies involving human participants were reviewed and approved by the Ethics Review Board of Sixth Affiliated Hospital of Sun Yat-sen University (2019ZSLYEC-80), and written informed consent was provided by the participants’ legal guardians. Written informed consent to participate in this study was provided by the participants’ legal guardian/next of kin.

## Author Contributions

SL and HH had full access to all the study data and took responsibility for data integrity and the accuracy of data analysis results. YC, FM, LQ, BL, HX, and YM contributed to the conception, study design, and data collection. All authors participated in the drafting of the article or its critical revision for important intellectual content and approved the publication of the final version of the manuscript.

## Conflict of Interest

The authors declare that the research was conducted in the absence of any commercial or financial relationships that could be construed as a potential conflict of interest.

## Footnotes

1 http://www.ncbi.nlm.nih.gov/geo/

2 http://string-db.org

3 https://www.gsea-msigdb.org/gsea/downloads.jsp

4 http://www.genome.jp/kegg/pathway.html

5 http://software.broadinstitute.org/gsea/index.jsp

## Abbreviations

AUC: area under the curve
BPD: bronchopulmonary dysplasia
GEO: Gene Expression Omnibus
GS: gene significance
MEs: module eigengenes
MM: module group members
PPI: protein–protein interaction
ROC: receiver operating characteristic
TOM: topological overlap matrix
WGCNA: weighted gene co-expression network analysis.
