# Supplementary material for: Weighted Gene Co-expression Network Analysis of Key Biomarkers Associated With Bronchopulmonary Dysplasia
Source: Front Genet. 2020 Sep 9;11:539292. doi: 10.3389/fgene.2020.539292 (PMC7509191; doi:10.3389/fgene.2020.539292)
Supplement: Supplementary file 2 [file Table_2.DOCX]

##data input##

library(WGCNA)

options(stringsAsFactors = FALSE)

enableWGCNAThreads(nThreads=8)

setwd("D:\\XX\\XX")

samples=read.csv('sample.txt',sep = '\t',row.names = 1)

expro=read.csv('expression matrix.txt',sep = '\t',row.names = 1)

dim(expro)

boxplot(expro)

#normalize#

library('limma')

expro=normalizeBetweenArrays(expro,method = "quantile")

boxplot(expro)

# 25%genes#

m.vars=apply(expro,1,var)

expro.upper=expro[which(m.vars>quantile(m.vars, na.rm = TRUE, probs = seq(0, 1, 0.25))[4]),]

dim(expro.upper)

datExpr=as.data.frame(t(expro.upper));

nGenes = ncol(datExpr)

nSamples = nrow(datExpr)

##SoftThreshold ##

powers = c(c(1:10), seq(from = 11, to=25, by=1))

sft = pickSoftThreshold(datExpr, powerVector = powers, verbose = 5)

par(mfrow = c(1,2));

cex1 = 0.9;

plot(sft$fitIndices[,1], -sign(sft$fitIndices[,3])*sft$fitIndices[,2],

xlab="Soft Threshold (power)",ylab="Scale Free Topology Model Fit,signed R^2",type="n",

main = paste("Scale independence"));

text(sft$fitIndices[,1], -sign(sft$fitIndices[,3])*sft$fitIndices[,2],

labels=powers,cex=cex1,col="red");

abline(h=0.90,col="red")

plot(sft$fitIndices[,1], sft$fitIndices[,5],

xlab="Soft Threshold (power)",ylab="Mean Connectivity", type="n",

main = paste("Mean connectivity"))

text(sft$fitIndices[,1], sft$fitIndices[,5], labels=powers, cex=cex1,col="red")

##One-step network construction and module detection##

net = blockwiseModules(datExpr, power = 23, maxBlockSize = 5500,

TOMType = "unsigned", minModuleSize = 50,

reassignThreshold = 0, mergeCutHeight = 0.25,

numericLabels = TRUE, pamRespectsDendro = FALSE,

saveTOMs = TRUE,

saveTOMFileBase = "AS-green-FPKM-TOM",

verbose = 3)

table(net$colors)

#sizeGrWindow(12, 9)

# Convert labels to colors for plotting

mergedColors = labels2colors(net$colors)

# Plot the dendrogram and the module colors underneath

plotDendroAndColors(net$dendrograms[[1]], mergedColors[net$blockGenes[[1]]],"Module colors",

dendroLabels = FALSE, hang = 0.03,

addGuide = TRUE, guideHang = 0.05)

##save###

moduleLabels = net$colors

moduleColors = labels2colors(net$colors)

table(moduleColors)

MEs = net$MEs;

geneTree = net$dendrograms[[1]];

save(MEs, moduleLabels, moduleColors, geneTree,

file = "AS-green-FPKM-02-networkConstruction-auto.RData")

nSamples = nrow(datExpr)

##clinic trait##

moduleLabelsAutomatic = net$colors

moduleColorsAutomatic = labels2colors(moduleLabelsAutomatic)

moduleColorsWW = moduleColorsAutomatic

MEs0 = moduleEigengenes(datExpr, moduleColorsWW)$eigengenes

MEsWW = orderMEs(MEs0)

modTraitCor = cor(MEsWW, samples, use = "p")

colnames(MEsWW)

modlues=MEsWW

modTraitP = corPvalueStudent(modTraitCor, nSamples)

textMatrix = paste(signif(modTraitCor, 2), "\n(", signif(modTraitP, 1), ")", sep = "")

dim(textMatrix) = dim(modTraitCor)

labeledHeatmap(Matrix = modTraitCor, xLabels = colnames(samples), yLabels = names(MEsWW), cex.lab = 0.9, yColorWidth=0.01,

xColorWidth = 0.03,

ySymbols = colnames(modlues), colorLabels = FALSE, colors = blueWhiteRed(50),

textMatrix = textMatrix, setStdMargins = FALSE, cex.text = 0.5, zlim = c(-1,1)

, main = paste("Module-trait relationships"))

##Export module genes##

module = "red";

# Select module probes

probes = names(datExpr)

inModule = (moduleColors==module);

modProbes = probes[inModule];

IMConn = softConnectivity(datExpr[, modProbes],power=23);

dat1=datExpr[inModule]

datExp_IMConn <-data.frame(IMConn,t(dat1))

datExp_IMConn=data.frame(datExp_IMConn)

write.table(datExp_IMConn, file = paste("Intramodule_connectivity-",module," .txt"),sep='\t')

module = "yellow";

# Select module probes

probes = names(datExpr)

inModule = (moduleColors==module);

modProbes = probes[inModule];

IMConn = softConnectivity(datExpr[, modProbes],power=23);

dat1=datExpr[inModule]

datExp_IMConn <-data.frame(IMConn,t(dat1))

datExp_IMConn=data.frame(datExp_IMConn)

write.table(datExp_IMConn, file = paste("Intramodule_connectivity-",module," .txt"),sep='\t')

##hub scatter plot##

modNames = substring(names(MEsWW), 3)

geneModuleMembership= as.data.frame(cor(datExpr, MEsWW, use ="p"))

Severity = as.data.frame(samples);

names(Severity) = "Severity"

geneTraitSignificance = as.data.frame(cor(datExpr, Severity, use = "p"));

module = "red"

column = match(module, modNames);

moduleGenes = moduleColors==module;

sizeGrWindow(7, 7);

par(mfrow = c(1,1));

verboseScatterplot(abs(geneModuleMembership[moduleGenes, column]),

abs(geneTraitSignificance[moduleGenes, 1]),

xlab = paste("Module Membership in", module, "module"),

ylab = "Gene significance for Severity",

main = paste("Module membership vs. gene significance\n"),

cex.main = 1.2, cex.lab = 1.2, cex.axis = 1.2, col = module)

abline(h=0.45,col="red")

abline(v=0.85,col="red")

module = "yellow"

column = match(module, modNames);

moduleGenes = moduleColors==module;

sizeGrWindow(7, 7);

par(mfrow = c(1,1));

verboseScatterplot(abs(geneModuleMembership[moduleGenes, column]),

abs(geneTraitSignificance[moduleGenes, 1]),

xlab = paste("Module Membership in", module, "module"),

ylab = "Gene significance for Severity",

main = paste("Module membership vs. gene significance\n"),

cex.main = 1.2, cex.lab = 1.2, cex.axis = 1.2, col = module)

abline(h=0.45,col="red")

abline(v=0.85,col="red")

MM= as.data.frame(cor(datExpr, MEsWW, use ="p"))

GS1 = as.data.frame(cor(datExpr, samples, use = "p"));

write.table (MM,file = "MM(all genes).xls",quote=F,sep='\t')

write.table (GS1,file = "GS1(all genes).xls",quote=F,sep='\t')
